# The ability of comorbidity indices to predict mortality in an orthopedic setting: a systematic review

**DOI:** 10.1186/s13643-021-01785-4

**Published:** 2021-08-18

**Authors:** Per Hviid Gundtoft, Mari Jørstad, Julie Ladeby Erichsen, Hagen Schmal, Bjarke Viberg

**Affiliations:** 1 Department of Orthopaedic Surgery and Traumatology, Lillebaelt Hospital, University Hospital of Southern Denmark, Sygehusvej 24, 6000 Kolding, Denmark; 2 Department of Orthopaedic Surgery, Aarhus University Hospital, Palle Juul-Jensens Boulevard 99, 8200 Aarhus N, Denmark; 3 Clinic of Orthopaedic Surgery Medical Center, Faculty of Medicine, University of Freiburg, Breisacher Straße 86b, 79110 Freiburg, Germany

**Keywords:** Comorbidity indices, Mortality, Orthopedics, Systematic review

## Abstract

**Background:**

Several comorbidity indices have been created to estimate and adjust for the burden of comorbidity. The objective of this systematic review was to evaluate and compare the ability of different comorbidity indices to predict mortality in an orthopedic setting.

**Methods:**

A systematic search was conducted in Embase, MEDLINE, and Cochrane Library. The search were constructed around two primary focal points: a comorbidity index and orthopedics. The last search were performed on 13 June 2019. Eligibility criteria were participants with orthopedic conditions or who underwent an orthopedic procedure, a comparison between comorbidity indices that used administrative data, and reported mortality as outcome. Two independent reviewers screened the studies using Covidence. The area under the curve (AUC) was chosen as the primary effect estimate.

**Results:**

Of the 5338 studies identified, 16 met the eligibility criteria. The predictive ability of the different comorbidity indices ranged from poor (AUC < 0.70) to excellent (AUC ≥ 0.90). The majority of the included studies compared the Elixhauser Comorbidity Index (ECI) and the Charlson Comorbidity Index (CCI). In-hospital mortality was reported in eight studies reporting AUC values ranging from 0.70 to 0.92 for ECI and 0.68 to 0.89 for CCI. AUC values were generally lower for all other time points ranging from 0.67 to 0.78. For 1-year mortality the overall effect size ranging from 0.67 to 0.77 for ECI and 0.69 to 0.77 for CCI.

**Conclusion:**

The results of this review indicate that the ECI and CCI can equally be used to adjust for comorbidities when analyzing mortality in an orthopedic setting.

**Trial registration:**

The protocol for this systematic review was registered on PROSPERO, the International Prospective Register of Systematic Reviews on 13 June 2019 and can be accessed through record ID 133,871.

**Supplementary Information:**

The online version contains supplementary material available at 10.1186/s13643-021-01785-4.

## Background

Research that uses data from registers and administrative databases often must deal with several potential sources of confounding factors, one of which is comorbidity. Comorbidity is all additional conditions, which have an effect on the patients’ conditions concomitant with the primary orthopedic condition. Therefore, taking into account and adjusting for comorbidity is often recommended when conducting research on mortality [1]. In an orthopedic setting, comorbidity is often relevant as it is associated with an increased risk of complications, re-operations, and mortality.

Adjustment for all known comorbidity variables is often not methodologically possible. Consequently, several comorbidity indices have been created to measure and adjust for the estimated burden of comorbidity. The Charlson Comorbidity Index (CCI) and the Elixhauser Comorbidity Index (ECI) are the most commonly used risk predictor tools [2]. CCI was developed and validated in 1987 [3] to predict risk of death within 1 year of hospitalization and since then different versions that uses administrative data have been created [4]. CCI was first adapted to ICD-codes by Deyo in 1992 and Romani in 1993 [5, 6]. Today, the most commonly used is the updated version by Quan et al. [4] that adapted CCI to be used with ICD-10 codes. The ECI was specifically developed using administrative data in 1998 [7] and with the CCI, Quan et al. also made an adaption of the ECI to the ICD-10 code.

The contents of these indices differ and have had numerous adaptations. Consequently, it has become difficult to identify which index to select when performing a risk assessment or when adjusting for comorbidity.

Previous systematic reviews on this topic have found several factors to be essential when selecting a comorbidity index. Yourkovich et al. [8] and Sharabiani et al. [9] concluded that the predictive ability of a comorbidity index primarily depends on the outcome and study population. However, no systematic review has investigated the validity of comorbidity indices in an orthopedic setting. The aim of this paper was to conduct a systematic review comparing the ability of different comorbidity indices to predict mortality in an orthopedic setting.

## Methods

This review is reported in accordance with the Preferred Reporting Items for Systematic Reviews and Meta-Analyses guidelines (PRISMA) and the PRISMA checklist can be found in Additional file 1 [10]. The protocol was registered in the International Prospective Register of Systematic Reviews (PROSPERO) on 13 June 2019 and can be accessed through record ID 133,871.

### Eligibility criteria

This systematic review included studies that compared the performance of different comorbidity indices in predicting mortality in an orthopedic setting.

Studies were included if they:

- Included participants with orthopedic conditions or participants who underwent an orthopedic procedure.
- Included a comparison of one comorbidity index with another comorbidity index.
- Included comorbidity indices that were based on administrative data (registers and databases).
- Reported mortality as an outcome.

Studies were excluded if they only included comorbidity indices that were validated only for specific diseases (e.g., the Cardiac-specific Comorbidity Index [11]). We did not have exclusion criteria in form of a minimum age limit nor for whether the study participants was in- or outpatients.

Primary outcome measures were defined as in-hospital, 30-day, 90-day, and 1-year mortality. Secondary outcome measures were not prespecified.

The language of included studies was restricted to articles written in English. No publication year, publication status, or study design limitations were imposed.

### Information sources

We searched the following databases: Ovid Embase Classic + Embase (1947–present), Ovid MEDLINE (R) all (1946–present), and the Cochrane Library. The last search was carried out on 2 May 2019. The reference list of included studies was screened by PHG and MJ for additional eligible studies. Grey literature was also searched through OpenGrey [12], the International Clinical Trial Registry Platform [13], and Orthopaedic Proceedings [14].

### Search

The search strategy was developed by the author team with assistance from librarians at the University of Southern Denmark and was primarily developed for Embase; thereafter, it was adapted for the other databases. The searches were constructed around two primary focal points: a comorbidity index and orthopedics. No language or publication year restrictions were used in the search. The following search terms were used to search Embase:

comorbidity ind*; comorbidity scor*; comorbidity scale*; exp Charlson Comorbidity Index; Charlson* adj2 (ind* or scale* or score*); Elixhauser*; Chronic Disease Score*; Rxrisk; Rx-risk; Rxrisk-v; Rx-risk-v; Medication-Based Disease Burden Index; exp orthopedics; orthop?edic*; exp orthopedic surgery; exp arthroplasty; arthroplast*; hemi-arthroplast*; hemiarthroplast*; exp osteoarthritis; osteoarthritis; exp arthroscopy; arthroscop*; exp fracture; fracture*; exp traumatology; traumatolog*; exp hip surgery; exp knee surgery; exp shoulder surgery; hip* or knee* or shoulder* adj3 (surg*).

A full description of the search strategies for Embase, MEDLINE, and the Cochrane Library can be found in the Additional file 2.

### Study selection

Eligibility assessment of the studies was performed independently in a blinded standardized manner by PHG and MJ using Covidence [15]. The two authors assessed the 5338 articles by screening title and subsequent reading the abstract of the studies and classified the studies as “eligible,” “not eligible,” or “perhaps eligible.” Following the independent assessment, the two authors then compared result. Disagreements between PHG and MJ were resolved by consensus and by consulting with the other authors.

### Data collection process

The data were extracted using a prespecified data collection form in Microsoft Excel.

Authors of three studies [16–18] were contacted for further information on the 95% confidence intervals (CIs). Bulöw et al. [16] and Kurichi et al. [18] responded and provided additional numerical data.

### Data items

The following information was extracted from each included study: (1) characteristics of participants (orthopedic diagnosis or surgical procedure); (2) type of comorbidity index; (3) relevant outcome measures (mortality); (4) database/register used; (5) data period; (6) country; (7) number of patients; (8) study design; and (9) relevant summary effect estimate.

### Risk of bias in individual studies

The risk of bias for each individual study was assessed by PHG and MJ using Risk Of Bias In Non-Randomized Studies of Interventions (ROBINS-I) [19].

### Summary measures

No summary effect measure was prespecified in the protocol. All 16 included studies reported the area under the curve (AUC) of receiver operating characteristic curve. Therefore, AUC was chosen as the primary effect estimate in this review; the AUC measures the predictive performance of a comorbidity index. The AUC value ranges from 0.5 to 1.0, where 0.5 indicates no predictive ability and 1.0 indicates perfect predictive ability. In general, an inexact guide can be used: 0.50: no discriminating ability, 0.60–0.69: poor accuracy, 0.70–0.79: fair accuracy, 0.80–0.89: good accuracy, and 0.90–1.00: excellent accuracy [20]. Confidence level of 95% is used when reporting AUC values.

### Data analysis

We originally intended to do a meta-analysis on the studies that compared CCI with ECI. However, due to the variation in the different versions and models of the comorbidity indices, differences in reported outcomes, and validity issues concerning the use of meta-analysis of predictive models [21] we omitted the meta-analysis and instead did a more narrative report of the studies.

## Results

### Study selection

**Fig. 1:**
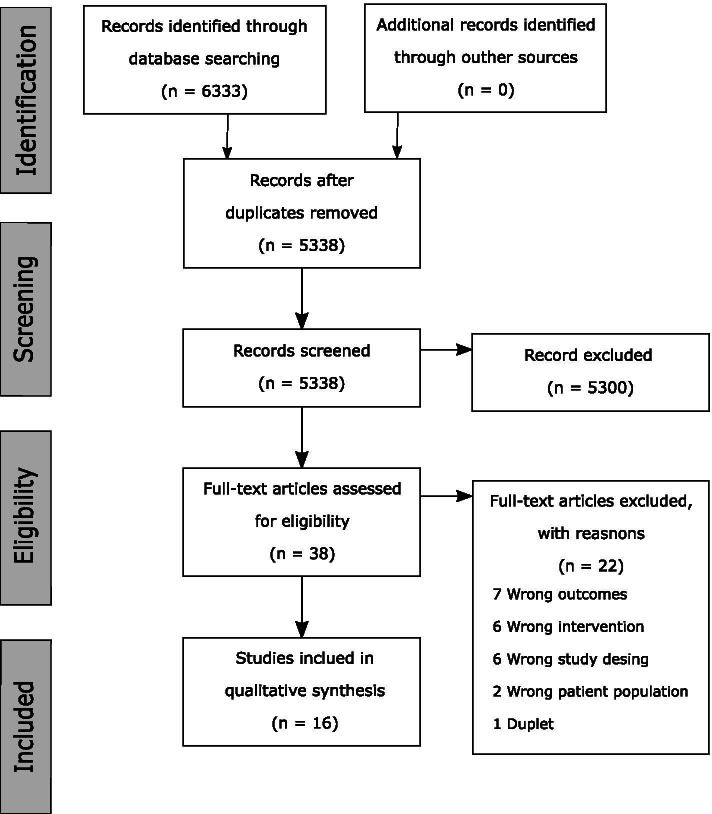
PRISMA flowchart. Legend: PRISMA flowchart of studies included in this review

The searches of the MEDLINE, Embase, and Cochrane Library databases provided a total of 6333 citations (Fig. 1). After adjusting for duplicates using Endnote and Covidence, 5338 citations remained. Of these, 5300 studies were discarded after reviewing the titles and abstracts (Fig. 1).

The full text of the remaining 38 citations was assessed. Sixteen studies met the inclusion criteria and were included in the systematic review (Table 1). No further studies were identified by screening the references of the included articles or searching the grey literature.

**Table 1:** Study characteristics for the included studies

Study	Period	Number of patients	Population	Country	Database/register	Compared indices	Mortality				
							*In-hospital*	*30 days*	*90 days*	*1 year*	*Other*
Boddaert et al. 2017 [22]	2009–2014	506	Hip fracture	France	Perioperative geriatric unit in a tertiary teaching hospital	CCI, Cumulative Illness Rating Scale, and POSPOM		Yes			6 months
Bulow et al. 2019 [16]	2005–2012	43 224	Arthroplasty for hip fracture	Sweden	Swedish Hip Arthroplasty Register	CCI Quan 2005, CCI Quan 2011, and ECI		Yes	Yes	Yes	2 years and 5 years
Bulow et al. 2017 [23]	1999–2012	120 836	Total hip arthroplasty	Sweden	Swedish Hip Arthroplasty Register	CCI Quan 2005, CCI Quan 2011, and ECI					5, 8, and 14 years
Inacio et al. 2016 [17]	2001–2002	30 820	Total hip and knee arthroplasty	Australia	Australian Government Department of Veterans' Affairs	CCI Quan 2005, ECI, RxRisk-V and combination of all three scores			Yes	Yes	
Kim et al. 2018 [24]	2002–2014	90 491	Total shoulder arthroplasty	USA	National Inpatient Sample	CCI Deyo 1992 and ECI	Yes				
Kurichi et al. 2007 [18]	2002–2003	2 375	Lower extremity amputations	USA	Veterans’ Health Administration databases	CCI Deyo 1992 and ECI				Yes	
Lopez-de-Andres et al. 2016 [25]	2003–2013	40 857	Lower extremity amputations	Spain	Spanish National Hospital database	CCI and ECI	Yes				
Menendez et al. 2014 [26]	1990–2007	14 007 813	Total Hip and Knee Arthroplasty, Spinal Fusion and Hip Fracture	USA	National Hospital Discharge Survey Database	CCI Deyo 1992 and ECI	Yes				
Menendez et al. 2015a [27]	2002–2011	387 973	Fracture proximal humerus	USA	Nationwide Inpatient Sample	CCI Deyo 1992 and ECI	Yes				
Menendez et al. 2015b [28]	1990–2007	111 564	Cervical spine fracture	USA	National Hospital Discharge Survey	CCI Deyo 1992 and ECI	Yes				
Menendez et al. 2015c [29]	1990–2007	33 194 141	Orthopedic surgery	USA	National Hospital Discharge Survey	CCI Deyo 1992, ECI and Combination of CCI and ECI	Yes				
Neuhaus et al. 2013 [30]	1990–2007	6 137 965	Hip fracture	USA	The National Hospital Discharge Survey	CCI Deyo 1992 vs. CCI Charlson 1994 vs. CCI Quan 2011	Yes				
Ondeck et al. 2018 [31]	–	49 738	Hip fracture	USA	National Inpatient Sample	CCI Quan 2005, ECI, and Modified Frailty Index	Yes				
Radley et al. 2008 [32]	1998–2000	43 811	Hip fracture	USA	Medicare enrollees	CCI Romano 1993, Clinical Classification Software and Iezzoni Index				Yes	
Toson et al. 2016 [33]	2008–2012	25 374	Hip fracture	Australia	The New South Wales Admitted Patient Data Collection	CCI Quan 2005, ECI, and Multipurpose Australian Comorbidity Scoring System	Yes	Yes		Yes	
Toson et al. 2016 [33	2001–2010	47 698	Hip fracture	Australia	The New South Wales Admitted Patient Data Collection	CCI Quan 2005 vs. CCI Sundararajan 04	Yes	Yes		Yes	

*CCI* Charlson Comorbidity Index, *ECI* Elixhauser Comorbidity Index

### Study characteristics

All of the studies were cohort studies (Table 1). All but one of the included studies [22] used various types of registers to gather the administrative data, with the National Hospital Discharge Survey being the most commonly used database. The data periods varied from 1990 to 2014. Nine of the studies were carried out in the USA. The number of patients included in each study ranged from 506 to 33,194,141.

Eight of the included studies compared only the ECI with the CCI [16, 18, 23–29]. In addition to these two indices, six other indices were also assessed: a Multipurpose Australian Comorbidity Scoring System, the Cumulative Illness Rating Scale, the Preoperative Score to Predict Postoperative Mortality, the Iezzoni Index, and the Clinical Classifications Software and Modified Frailty Index.

Regarding the outcome measures, in-hospital mortality was the most commonly reported outcome, followed by 1-year and 30-day mortality.

### Risk of bias within studies

Most studies were evaluated as having a moderate risk of bias. Two studies were evaluated to have serious risk of bias due to either confounding or bias in reported results (Table 2).

**Table 2:** Assessment of risk of bias using ROBINS-I assessment tool

**Pre-intervention and at-intervention domains**				**Post-intervention domains**				
*Study*	*Confounding*	*Selection*	*Classification of intervention*	*Deviation from intended intervention*	*Missing data*	*Measurement of outcomes*	*Reported result*	*Overall*
Boddaert et al. 2017 [22]	Serious	Low	Moderate	Low	Low	Low	Moderate	Serious
Bulow et al. 2019 [16]	Moderate	Low	Moderate	Low	Low	Low	Moderate	Moderate
Bulow et al. 2017 [23]	Moderate	Low	Moderate	Low	Low	Low	Moderate	Moderate
Inacio et al. 2016 [17]	Moderate	Low	Moderate	Low	Low	Low	Moderate	Moderate
Kim et al. 2018 [24]	Moderate	Low	Moderate	Low	Low	Low	Moderate	Moderate
Kurichi et al. 2007 [18]	Moderate	Low	Moderate	Low	Low	Low	Serious	Serious
Lopez-de-Andres et al. 2016 [25]	Moderate	Low	Moderate	Low	Low	Low	Moderate	Moderate
Menendez et al. 2014 [26]	Moderate	Low	Moderate	Low	Low	Low	Moderate	Moderate
Menendez et al. 2015a [27]	Moderate	Low	Moderate	Low	Low	Low	Moderate	Moderate
Menendez et al. 2015b [28]	Moderate	Low	Moderate	Low	Low	Low	Moderate	Moderate
Menendez et al. 2015c [29]	Moderate	Low	Moderate	Low	Low	Low	Moderate	Moderate
Neuhaus et al. 2013 [30]	Moderate	Low	Moderate	Low	Low	Low	Moderate	Moderate
Ondeck et al. 2018 [31]	Moderate	Low	Moderate	Low	Low	Low	Moderate	Moderate
Radley et al. 2008 [32]	Moderate	Low	Moderate	Low	Low	Low	Moderate	Moderate
Toson et al. 2016 [33]	Moderate	Low	Moderate	Low	Low	Low	Moderate	Moderate
Toson et al. 2016 [33]	Moderate	Low	Moderate	Low	Low	Low	Moderate	Moderate

### Results of individual studies

The 16 included studies varied markedly regarding the type and version of comorbidity indices assessed, and the follow-up time, which ranged from in-hospital to 14 years (Table 1). In eight studies (Bülow et al. 2019 [16], Bülow et al. 2017 [23], Menendez et al. 2014 [26], Menendez et al. 2015 [28], Menendez et al. 2015 [29], Ondeck et al. 2008 [31], Radley et al. 2008 [32], and Toson et al. 2016 [33), the comorbidity indices was compared to a base model, comprising of demographic variables (e.g., age and sex) and in two of those, the base model outperformed both CCI and ECI.

In nine studies, the comorbidity indices were combined with demographic variables (e.g., age and sex). In all nine studies, this combination increased the predictive power.

#### Comparison of the Elixhauser and Charlson Comorbidity Indices

The CCI and ECI were analyzed and compared in 12 studies (Table 3).

**Table 3:** Area under the curve (AUC) values for Elixhauser Comorbidity Index (ECI) and Charlson Comorbidity Index (CCI) for studies reporting

Study	In-hospital		30 days		1 year	
	CCI	ECI	CCI	ECI	CCI	ECI
Bulow et al. 2019 [16]			0.69 [0.68; 0.69]	0.67 [0.67; 0.68]	0.69 [0.68; 0.69]	0.67 [0.66; 0.67]
Inacio et al. 2016 [17]					0.77	0.77
Kim et al. 2018 [24]	0.807 [0.753; 0.862]	0.854 [0.806; 0.903]				
Kurichi et al. 2007 [18]					0.68 [0.608; 0.715]	0.70 [0.657; 0.793]
Lopez-de-Andres et al. 2016 [25]	0.89 [0.88; 0.90]	0.92 [0.90; 0.93]				
Menendez et al. 2014 [26]	0.794 [0.792; 0.796]	0.845 [0.844; 0.847]				
Menendez et al. 2015a [27]	0.83 [0.83; 0.84]	0.86 [0.86; 0.86]				
Menendez et al. 2015b [28]	0.786 [0.771; 0.801]	0.840 [0.828; 0.853]				
Menendez et al. 2015c [29]	0.823 [0.819; 0.828]	0.852 [0.848; 0.856]				
Neuhaus et al. 2013 [30]	0.77					
Ondeck et al. 2018 [31]	0.682 [0.665; 0.698]	0.697 [0.680; 0.713]				
Radley et al. 2008 [32]	0.68					
Toson et al. 2015 [33	0.779 [0.767; 0.791]	0.777 [0.766; 0.789]	0.767 [0.757; 0.779]	0.731 [0.719; 0.743]	0.734 [0.727; 0.742]	0.705 [0.697; 0.712]
Toson et al. 2016 [33]	0.759 [0.751; 0.767]		0.753 [0.745; 0.761]		0.734 [0.729; 0.739]	

In-hospital, 30-, or 1-year mortality

Eight of these studies investigated the in-hospital mortality [24, 25, 27–33]. The AUC values of the ECI ranged from 0.78 to 0.92 when focusing on adjusted values only, reflecting fair to excellent predictive ability. The CCI adjusted values were similar, ranging from 0.78 to 0.89.

Two studies reported on the 30-day mortality [16, 33] and found that the predictive ability of both indices was quite low, ranging from poor to fair (0.67–0.767).

Regarding 1-year mortality, four studies reported on both CCI and ECI for this time point [16–18, 33], and the two indices had similar AUC values, ranging from poor to fair (0.67–0.77).

Long-term mortality (2, 5, 8, and 14 years) was assessed in two studies [16, 23]. The CCI and ECI showed similar poor to fair predictive power for long-term mortality (0.66–0.76).

#### Other indices

In addition to the CCI and ECI, six other indices were identified. None of these six indices were assessed in more than one study. Most of the other indices were compared with the CCI and/or ECI, and in general they performed equally good or better, with the exception of the RxRisk-V and the Modified Frailty Index, which had AUC values that were slightly lower than those of the CCI and ECI [17, 31].

The Multipurpose Australian Comorbidity Scoring System demonstrated good discriminating ability in predicting in-hospital (AUC 0.81 (0.80–0.82)) and 30-day mortality (AUC 0.80 (CI 0.79–0.81)), but only fair prediction of 1-year mortality (AUC 0.77 (CI 0.76–0.77)) [33].

It is also worth recognizing that two studies assessed more inclusive comorbidity measure that was generated by combining comorbidities from the ECI and CCI (Menendez et al. [29] or the ECI, CCI, and RxRisk-V [17]. Both studies showed that the combination of comorbidity indices outperformed the ECI and the CCI.

## Discussion

### Summary of evidence

This systematic review summarizes the evidence on how different comorbidity indices compare to one another in predicting mortality in an orthopedic setting. Most studies compare the ECI and CCI and found no clear difference in the predictive ability of these two indices. Six studies compared other comorbidity indices to the CCI and ECI and four of those performed equally good or better. For all comorbidity indices, adjusting for demographic factors together with comorbidities generally provided better predictive ability.

We found no clear difference in the predictive ability of the ECI and CCI. Both have good to excellent predictive ability for in-hospital mortality, but only poor to fair performance for all other time points. As the ECI and CCI comprise of several of the same comorbidity conditions this result might not be surprising. However, these findings are inconsistent with other studies on this topic. Yurkovich et al. [8] conducted a systematic review on comorbidity indices using administrative data and concluded that the ECI consistently outperformed the CCI in predicting both short- and long-term mortality. Sharabiani et al. [9] conducted a systematic review on comorbidity indices and found that comorbidity adjustment methods showed better prediction for long-term (> 30 days) than short-term (< 30 days) mortality. However, neither of these reviews specified a particular patient setting. Therefore, one hypothesis to explain this discrepancy of results can be the differences in the patient settings. This corresponds well with the statement by Yurkovich et al. [8] that the predictive ability of the comorbidity indices primarily depends on the outcome and study population. Comorbidity indices might not perform as well in an orthopedic setting compare to other settings. Therefore, any difference between comorbidity indices might not be evident. Another difference to consider is that the current review included fewer studies than the two previously mentioned reviews.

The Multipurpose Australian Comorbidity Scoring System [33], the study by Menendez et al. [29], and the study Inacio et al. [17] were all characterized by including a large number of comorbidities and all three performed well. The systematic review by Sharabiani et al. [9] found the same association. However, the evidence is not sufficiently extensive at this point to make a clear recommendation to use these newly developed comorbidity indices. As this study shows that the predictive ability of comorbidity indices are very similar, the choice of which comorbidity index to use in adjusting of comorbidity must to a large extend depend on other variables. The most correct method of choosing which comorbidity index to use is to do a validation of the indices for the study population of interest. However, this is a time- and labor-consuming work. Other factors might also play a role, such as comparability to previous studies, which use a specific index, and which comorbidity variables that are available in the relevant register.

Since comorbidity is associated with mortality, one would presumably expect that adjustment for comorbidity would increase the predictive ability considerably. This was not always evident in this review. One challenge with using administrative data to gather information on comorbidities is the potential underreporting in administrative databases and coding practices. Several studies have shown that administrative data can be an unreliable source [34, 35]. This might explain the varying and sometimes poor predictive ability of the comorbidity indices. The choice of which administrative database to use is of importance, and research on this matter is limited.

### Limitations

There are several limitations of this work. This review was limited by heterogeneity in the included studies. For instance, the CCI was assessed as one index all together when in fact it includes all of its subtypes, with various amounts of comorbidities (ranging from 12 to 19) and different weighting algorithms. Initially, we wanted to do an exploratory subgroup analysis for the subtypes of CCI and ECI, but we were not able to define all of them due to insufficient descriptions in each study.

Additionally, although the patient population was more specific than in previous systematic reviews on this topic [8, 9], it could still be classified as broad. We can assume, for instance, that patients undergoing arthroplasty surgery for osteoarthritis often differ significantly from patients with hip fracture. Therefore, it is debatable how comparable these patient groups are.

Further, we were not able to define in-hospital mortality. Therefore, this time period could vary markedly between studies and might cause some misinterpretation of the results.

Finally, at the moment, the overall evidence is not sufficiently extensive to determine the appropriate index for every mortality time point.

## Conclusions

The results of this review indicate that the ECI and CCI can equally be used to adjust for comorbidities when analyzing in-hospital mortality in an orthopedic setting where they have fair to excellent predictive ability. However, in general, both indices have poor to fair AUC values for 30-day, 90-day, and 1-year mortality.

## Supplementary Information

**Additional file 1.** PRISMA checklist.

**Additional file 2.** Search strategy for A) Embase (Ovid), B) Medline (Ovid) and C) Cochrane Library.

## Data Availability

Not applicable.

## Abbreviations

AUC: Area under the curve
CCI: Charlson Comorbidity Index
CI: Confidence intervals
ECI: Elixhauser Comorbidity Index
ROBINS-I: Risk Of Bias In Non-Randomized Studies of Interventions
