# Supplementary material for: The ability of comorbidity indices to predict mortality in an orthopedic setting: a systematic review
Source: Syst Rev. 2021 Aug 18;10:234. doi: 10.1186/s13643-021-01785-4 (PMC8375166; doi:10.1186/s13643-021-01785-4)
Supplement: Supplementary file 2 — Additional file 2. Search strategy for A) Embase (Ovid), B) Medline (Ovid) and C) Cochrane Library. [file 13643_2021_1785_MOESM2_ESM.docx]

**dix**

**Additional file 2**

**Search strategy** for A) Embase (Ovid), B) Medline (Ovid) and C) Cochrane Library

**A)** Embase (Ovid)

|  | Search term | Hits 02.05.19 |
| --- | --- | --- |
|  | comorbidity ind*.mp. | 21309 |
|  | comorbidity scor*.mp. | 3983 |
|  | comorbidity scale*.mp. | 79 |
|  | exp Charlson Comorbidity Index/ | 15086 |
|  | Charlson* adj2 (ind* or scale* or score*).mp. | 21275 |
|  | Elixhauser*.mp. | 1283 |
|  | chronic disease score*.mp. | 249 |
|  | Rxrisk.mp. or Rx-risk.mp | 85 |
|  | Rxrisk-v.mp or Rx-risk-v.mp | 49 |
|  | Medication-Based Disease Burden Index.mp. | 8 |
|  | 1 or 2 or 3 or 4 or 5 or 6 or 7 or 8 or 9 or 10 | 26966 |
|  | exp orthopedics/ | 26897 |
|  | orthop?edic*.mp. | 155 678 |
|  | exp orthopedic surgery/ | 482092 |
|  | exp arthroplasty/ | 68573 |
|  | arthroplast*.mp. | 97976 |
|  | hemi-arthroplast*.mp. or hemiarthroplast*.mp | 4216 |
|  | exp osteoarthritis/ | 129024 |
|  | osteoarthritis.mp. | 139376 |
|  | exp arthroscopy/ | 29272 |
|  | arthroscop*.mp. | 44795 |
|  | exp fracture/ | 308069 |
|  | fracture*.mp. | 404774 |
|  | exp traumatology/ | 11522 |
|  | traumatolog*.mp. | 17579 |
|  | exp hip surgery/ | 27821 |
|  | exp knee surgery/ | 41982 |
|  | exp shoulder surgery/ | 6666 |
|  | hip* or knee* or shoulder* adj3 (surg*).mp. | 38184 |
|  | 12 or 13 or 14 or 15 or 16 or 17 or 18 or 19 or 20 or 21 or 22 or 23 or 24 or 25 or 26 or 27 or 28 or 29 | 976 995 |
|  | 11 and 30 | 2529 |

**B)** Medline (Ovid)

|  | Search term | Hits 02.05.19 |
| --- | --- | --- |
|  | comorbidity ind*.mp. | 6116 |
|  | comorbidity scor*.mp. | 1661 |
|  | comorbidity scale*.mp. | 36 |
|  | Charlson* adj2 (ind* or scale* or score*).mp. | 7065 |
|  | Elixhauser*.mp. | 440 |
|  | chronic disease score*.mp. | 145 |
|  | Rxrisk.mp. or Rx-risk.mp | 42 |
|  | Rxrisk-v.mp or Rx-risk-v.mp | 30 |
|  | Medication-Based Disease Burden Index.mp. | 4 |
|  | 1 or 2 or 3 or 4 or 5 or 6 or 7 or 8 or 9 | 9554 |
|  | exp orthopedics/ | 19960 |
|  | orthop?edic*.mp. | 111328 |
|  | exp arthroplasty/ | 62263 |
|  | arthroplast*.mp. | 78791 |
|  | hemi-arthroplast*.mp. or hemiarthroplasty*.mp | 3114 |
|  | exp osteoarthritis/ | 58410 |
|  | osteoarthritis.mp. | 80940 |
|  | exp arthroscopy/ | 21938 |
|  | arthroscop*.mp. | 33159 |
|  | exp fracture dislocation/ | 2049 |
|  | exp fracture fixation/ | 58246 |
|  | exp fracture healing/ | 12516 |
|  | fracture*.mp. | 287142 |
|  | exp traumatology/ | 3320 |
|  | traumatolog*.mp. | 8248 |
|  | hip* or knee* or shoulder* adj3 (surg*).mp. | 21291 |
|  | 11 or 12 or 13 or 14 or 15 or 16 or 17 or 18 or 19 or 20 or 21 or 22 or 23 or 24 or 25 or 26 | 529 501 |
|  | 10 and 27 | 934 |

**C)** Cochrane Library

|  | Search term | Hits 02.05.19 |
| --- | --- | --- |
|  | (comorbidity ind*) | 12 029 |
|  | (comorbidity scor*) | 6376 |
|  | (comorbidity scale*) | 5352 |
|  | (Rx-risk) | 1 |
|  | (Medication-Based Disease Burden Index) | 9 |
|  | (chronic disease score*) | 18 245 |
|  | (Elixhauser) | 29 |
|  | ((Charlson* NEAR/2 (ind* or scale* or score*))) | 783 |
|  | #1 or #2 or #3 or #4 or #5 or #6 or #7 or #8 | 25 307 |
|  | MeSH descriptor: [Orthopedics] explode all trees | 336 |
|  | (orthopedic*) | 11 211 |
|  | MeSH descriptor: [Arthroplasty] explode all trees | 4557 |
|  | (arthroplast*) | 10 801 |
|  | (hemiarthroplast*) | 428 |
|  | MeSH descriptor: [Osteoarthritis] explode all trees | 6344 |
|  | (osteoarthritis) | 15 646 |
|  | MeSH descriptor: [Arthroscopy] explode all trees | 1374 |
|  | (arthroscop*) | 4708 |
|  | (fracture*) | 20 973 |
|  | MeSH descriptor: [Traumatology] explode all trees | 29 |
|  | (traumatolog*) | 2877 |
|  | ((hip* or knee* or shoulder* NEAR/3 (surg*))) | 52 109 |
|  | #10 or #11 or #12 or #13 or #14 or #15 or #16 or #17 or #18 or #19 or #20 or #21 or #22 | 78 221 |
|  | #9 and #23 | 2870 |
